# Clinical Impact of Down-Regulated Plasma miR-92a Levels in Non-Hodgkin's Lymphoma

**DOI:** 10.1371/journal.pone.0016408

**Published:** 2011-02-24

**Authors:** Kazuma Ohyashiki, Tomohiro Umezu, Sei-ichiro Yoshizawa, Yoshikazu Ito, Michiyo Ohyashiki, Hisashi Kawashima, Masami Tanaka, Masahiko Kuroda, Junko H. Ohyashiki

**Affiliations:** 1 Hematology Division, First Department of Internal Medicine, Tokyo Medical University, Tokyo, Japan; 2 Department of Pediatrics, Tokyo Medical University, Tokyo, Japan; 3 Department of Molecular Pathology, Tokyo Medical University, Tokyo, Japan; 4 Intractable Disease Research Center, Tokyo Medical University, Tokyo, Japan; Institut Jacques Monod, France

## Abstract

**Background:**

We undertook a study to evaluate the clinical relevance of miR-92a in plasma obtained from non-Hodgkin's lymphoma (NHL) patients, because the miR-17-92 polycistronic miRNA cluster plays a crucial role in lymphomagenesis and affects neo-angiogenesis.

**Methodology/Principal Findings:**

Plasma miR-92a values in NHL were extremely low (<5%), compared with healthy subjects (P<.0001), irrespective of lymphoma sub-type. The very low plasma level of miR-92a increased in the complete response (CR) phase but did not reach the normal range, and the plasma level was lower again in the relapse phase. Patients in CR or CR unconfirmed with a plasma miR-92a level of less than the cut-off level showed a significantly high relapse rate compared with patients with normalized plasma miR-92a level.

**Conclusions/Significance:**

The current results therefore indicate that the plasma miR-92a value could be a novel biomarker not only for diagnosis but also for monitoring lymphoma patients after chemotherapy.

## Introduction

MicroRNAs (miRNAs) are small non-coding RNAs that range from 18 to 24 nucleotides in length and regulate gene expression by the RNA-induced silencing complex, resulting in a reduction of the translation and stability of target messenger RNAs [1]–[3]. Recent studies have shown the presence of miRNA alterations in various human cancers, suggesting that miRNAs play important roles in carcinogenesis and disease progression [4]–[6]. The miR-17-92 cluster is a polycistronic miRNA gene, encoding six miRNAs (miR-17, miR-18a, miR-19a, miR-19b, miR-20a, and miR-92). Several lines of evidence have suggested the importance of the miR-17-92 cluster in immune cell development, as well as in tumorigenesis in lymphoid tissue [7]–[9]. In mouse experiments, *MYC* regulates the miR-17-92 cluster [7], [9], [10] whose ectopic expression in lymphocytes is associated with severe lymphoproliferative disorders owing to the targeting of *BIM*, a proapoptotic member of the anti-apoptotic *BCL2* family [9]. In humans, over-expression of the miR-17-92 cluster has been demonstrated in various neoplasms, including lymphoma, multiple myeloma, ovarian cancer, and lung cancer [11]–[15], perhaps owing to possible induction of anti-apoptosis.

Activation of the miR-17-92 pathway triggered by *MYC* may in part affect augmentation of tumor angiogenesis: Dews et al. demonstrated that *MYC*-transformed colonocyte-bearing mice showed an enhancement of tumor neo-angiogenesis with down-regulated thrombospodin-1 and connective growth factor [16]. In contrast, members of the miR-17-92 cluster have recently been identified to have cell intrinsic anti-angiogenic function in human endothelial cells [17]. MiR-92a is known to control angiogenesis in an ischemic mice model [18], and involvement of angiogenesis is crucial in the progression of lymphoma or multiple myeloma patients [19], [20].

Recently, studies have demonstrated that circulating miRNAs can be detected in the plasma and serum of healthy subjects and cancer patients [21]–[24], including those with lymphoma [25]. Therefore, deviations in levels of certain miRNAs detected in the plasma of cancer patients could be a useful and non-invasive marker to detect cancer development. We have recently demonstrated that miR-92a dramatically decreased in the plasma of acute leukemia patients [26]. This evidence allows us to examine the miR-92a expression levels in plasma obtained from patients with lymphoma and clarify the clinical relevance of plasma levels of miR-92a, given that the miR-17-92 cluster is crucial in lymphomagenesis.

## Results

### Microarray analysis of miRNAs in the plasma of lymphoma

**Figure 1:**
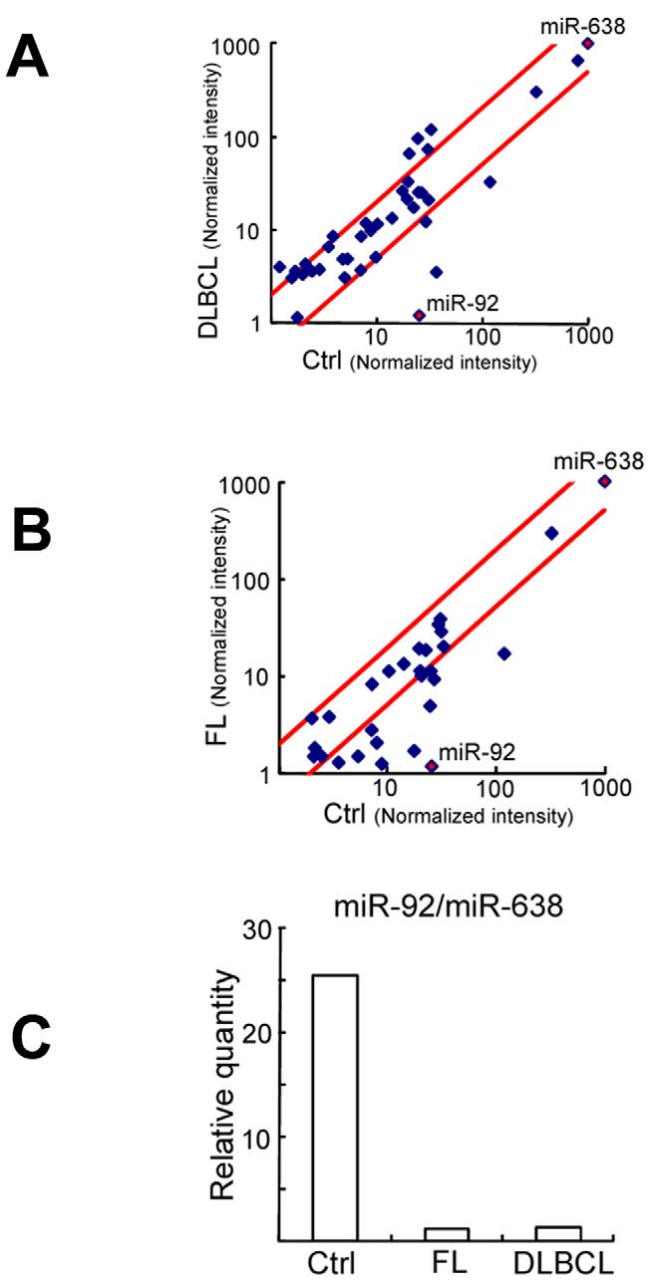
Comparison of miR expressions in the plasma of normal subjects and non-Hodgkin's lymphoma patients by MiRNA microarray. Comparison of normalized signal intensities of various miRs in plasmas (miR-638 adjusted to 1000). X axis represents healthy control (Ctrl), and Y axis represents diffuse large B-cell lymphoma (DLBCL) (A) or follicular lymphoma (FL) (B). Red squares show miR-638 (control) and miR-92a. (C) Comparison of the ratio of miR92/miR-638 signal intensity in the plasma of Ctrl, FL, and DLBCL.

To identify the differentially expressed miRNAs in the plasmas of NHL patients, we first screened NHL samples using microarrays. Experiments were performed with total RNA isolated from the plasma of diffuse large B-cell lymphoma (DLBCL) samples (n = 4), follicular lymphoma (FL) samples (n = 4), and healthy controls (n = 8). Total RNA in each group was pooled and used for microarray experiments. The signals of miR-638 from normal subjects constituted the top rank, regardless of sex or age [26]. Our data showed that the miR-638 probe had the highest intensity in all DLBCL (**Figure 1A**) and FL (**Figure 1B**) samples; therefore, we applied miR-638 signal values for normalization. By contrast, we found that signal intensities of miR-92 were dramatically reduced in both FL and DLBCL samples compared with healthy controls (**Figure 1C**).

### Validation of plasma miR-92a expression level

**Figure 2:**
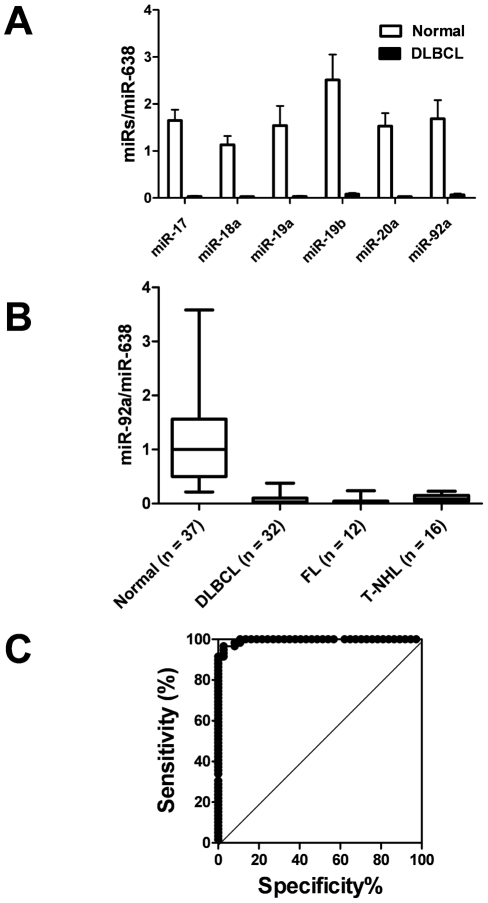
Plasma miR-92a expression level and ROC curve in patients with non-Hodgkin's lymphoma by quantitative real-time PCR. (A) Plasma miR-17-92a expression levels in normal subjects (open bars: Normal: n = 5) and diffuse large B-cell lymphoma (solid bars: DLBCL: n = 13), indicating that all of these miR-17-92a polycistronic miRs were remarkably decreased in plasma obtained from DLBCL patients. Boxes show 95 percentile confidence intervals and lines indicate the range of each miR values. (B) Plasma miR-92a value (miR-92a/miR-638) in healthy subjects, and in patients with diffuse large B-cell lymphoma, follicular lymphoma, and T-cell non-Hodgkin's lymphoma (T-NHL) at diagnosis. Boxes show 95 percentile confidence intervals; lines indicate the range of miR-92a values and also show significantly low levels of plasma miR-92a in various lymphomas, compared with healthy subjects (*P*<.0001). (C) ROC curve analysis using GraphPad Prism 5.0 software. The cut-off level of plasma miR-92a/miR-638 in all non-Hodgkin's lymphoma patients (n = 60) was 0.2165; the sensitivity was 91.85% (95% CI: 81.32% to 97.19%) and specificity was 97.3% (95% CI: 85.84% to 99.93%).

The results obtained from the microarray analysis described the stable detection and reproducible results of the miR-638 expression level in plasma. Since we could not detect plasma U6B, which is commonly used as an internal standard for miRNA expression analysis in cells, we used miR-638 as a reference in each sample, as previously reported [26], [27]. From these results, we obtained expression levels of miR-92a (i.e., miR-92a/miR-638), and took the result to be the miR-92a plasma level. Moreover, we confirmed that plasma miR-92a expression could represent miR-17-92a polycistronic miRNA in lymphoma patients (**Figure 2A**). We were unable to detect plasma miR-638 expression in two lymphoma patients by the quantitative real-time PCR method; therefore, we regarded these two specimens as PCR failures and excluded their results from the statistical analysis. We determined miR-92a levels in 37 plasma specimens from healthy subjects and performed statistical analysis using 144 specimens from NHL patients.

### Decreased plasma miR-92a levels at diagnosis of lymphoma

We found significantly lower plasma level values of miR-92a at diagnosis, compared with those of normal subjects, regardless of lymphoma sub-type (*P*<.0001; **Figure 2B**). The expression level of plasma miR-92a in patients with NHL was less than 5% that of healthy volunteers. Among lymphoma sub-types, the plasma miR-92a level did not differ between DLBCL and FL (*P* = .927) at diagnosis; however, the plasma miR-92a level in T-cell lymphoma tended to be higher than that in FL (*P* = .014) or DLBCL (*P* = .057) (**Table S1**).

We determined the sensitivity and specificity using receiver operating characteristic (ROC) curve analysis. The area under the ROC curve was 0.9954. When the cut-off level of plasma miR-92a/miR-638 in all 60 NHL patients at diagnosis was 0.2165, the sensitivity was 91.85% (95% confidence interval [CI]: 81.32% to 97.19%), and the specificity was 97.3% (95% CI: 85.84% to 99.93%) (**Figure 2C**). We then analyzed the 0.2165 cut-off value of miR-92a/miR-638 to determine clinical significance.

### Clinical relevance of plasma miR-92a at various states

**Figure 3:**
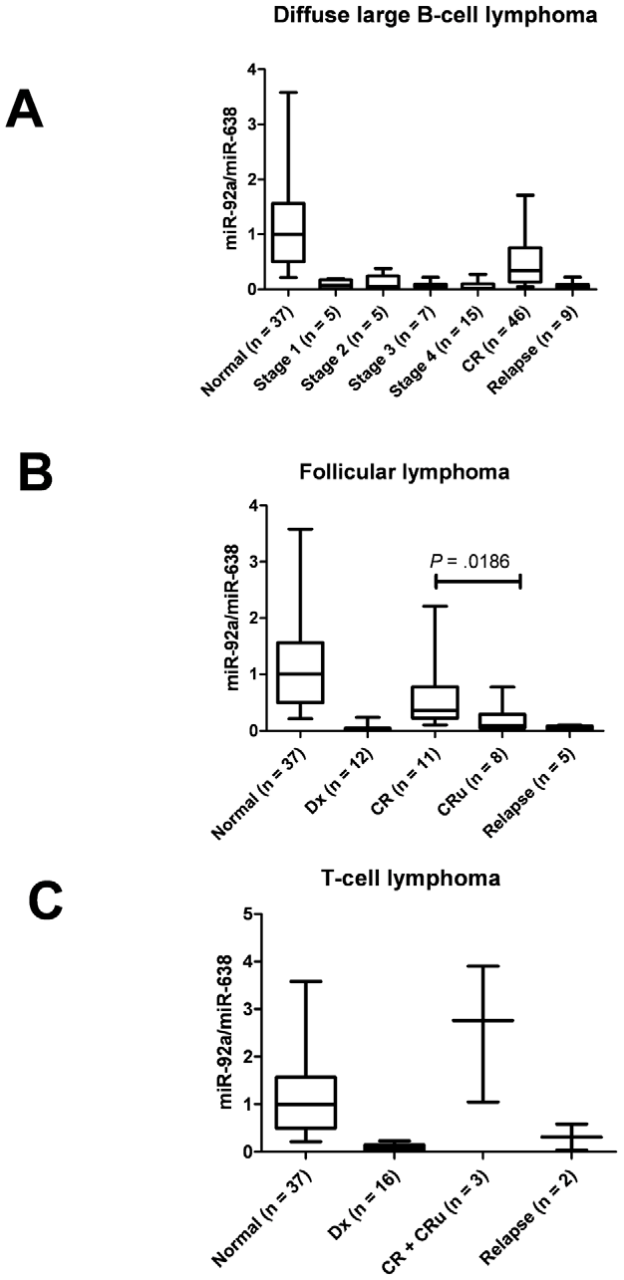
Plasma miR-92a value (miR-92a/miR-638) in patients with non-Hodgkin's lymphoma at various stages. (A) Plasma miR-92a value (miR-92a/miR-638) in patients with diffuse large B-cell lymphoma at the time of diagnosis, in the relapse phase, and in the complete remission (CR) state. Decreased plasma miR-92a values recovered in the CR phase (*P*<.0001) but were still significantly lower than in normal subjects (*P*<.0001). Plasma miR-92a levels at relapse decreased again and the levels were similar to those at diagnosis. (B) Plasma miR-92a value (miR-92a/638) in patients with follicular lymphoma at the time of diagnosis (Dx), in the relapse phase, and in the CR or complete remission unconfirmed (CRu) states. Patients with CRu status had significantly lower plasma miR-92a/miR-638 values compared with those who had CR (*P* = .0186 by the Mann-Whitney test). (C) Plasma miR-92a values (miR-92a/miR-638) in patients with T-cell non-Hodgkin's lymphoma at the time of diagnosis, relapse phase, and complete remission (CR and CRu).

The plasma miR-92a level at diagnosis did not differ according to clinical stage among DLBCL patients (**Figure 3A**). Plasma miR-92a levels in DLBCL patients in CR increased significantly compared with the level at diagnosis (*P*<.0001) but were still significantly lower than those in healthy individuals (*P*<.0001). In the relapse phase, plasma miR-92a levels again decreased (*P*<.0001), and these levels were similar to those at the time of diagnosis (*P* = .8132; **Figure 3A**). Similarly, a significantly low level of plasma miR-92a was noted in patients with FL at the time of diagnosis (*P*<.0001), but it partially normalized after the patients obtained CR and decreased again in the relapse phase. The plasma miR-92a level in FL patients classified as CRu was decreased compared with that of CR patients (*P* = .0186; **Figure 3B**). At relapse, the plasma miR-92a level in FL patients was significantly decreased compared with that of CR plus CRu patients (*P* = .0089). The fluctuation pattern of plasma miR-92a detection levels in relation to clinical status in T-cell NHL was similar to that of DLBCL and FL (**Figure 3C**).

### Relapse risk analysis

Of the patients with CR (including CRu in FL) at the time of examination of plasma miR-92a who had been followed up for more than 6 months and who did not receive any hematopoietic stem cell transplantation, a significant higher relapse rate was evident in patients showing low plasma miR-92a levels (below the 0.2165 cut-off level) compared with those with normalized plasma miR-92a (above 0.2165). DLBCL patients with low plasma miR-92a relapsed significantly compared with those with normal range plasma miR-92a (5/12 versus 1/22; *P* = .007 by the chi-square test; Table 1). The odds ratio of relapsed DLBCL with a low miR-92a value was 15 (95% CI: 1.486 to 151.4) by univariate analysis. Although the number of study patients with DLBCL was small, the link between low plasma miR-92a values in CR and the relapse rate was notable, but not significant, in patients with advanced clinical stage (III and IV) at diagnosis (3/5 versus 2/13; *P* = .0584) and in those with higher International Prognostic Scores (IPI) (2/4 versus 1/10; *P* = .0994). Multivariate analysis revealed that only down-regulated plasma miR-92a was significantly lower in relapsed DLBCL patients (*P* = .02). In addition, the corrected odds ratio was 16.232 (95% CI: 1.562 to 168.725), compared with that of the normalized plasma miR-92a value determined at the clinical CR phase (**Table 1**).

**Table 1:** Statistical analysis of relapse in diffuse large B cell lymphoma patients whose miR-92a levels were determined at complete response phase.

			univariate			multivariate		
Factors at diagnosis	No. (n = 34*)	Relpased (n = 6)	*P* value	odds ratio	95% CI	*P* value	Corrected odds ratio	95% CI
Sex								
male	13	1	.231	0.267	0.02743 to 2.592	.833	1.3037	0.066 to 8.971
female	21	5						
Age								
≥60 years	20	4	1	1.5	0.2346 to 9.593	.50	0.418	0.03 to 5.287
<60 years	14	2						
LDH								
Elevated	16	4	.289	2.667	0.4170 to 17.05	.673	1.625	0.170 to 15.522
Normal	18	2						
B symptoms								
Yes	16	3	.874	1.154	0.1976 to 6.738	.853	0.730	0.026 to 20.544
No	18	3						
Clinical stage								
III+IV	18	5	.18	5.769	0.5946 to 55.98	.062	23.189	0.858 to 629.583
I+II	16	1						
International Prognostic Index								
high + high-int	14	3	.628	1.545	0.2629 to 9.085	.972	0.943	0.038 to 23.744
low + low int	20	3						
Plasma miR-92a								
Low (<0.2165)	12	5	.007	15	1.486 to 151.4	.020	16.232	1.562 to 168.725
Normal (≥0.2165)	22	1						

Abbreviations: CI, confidence interval; LDH, lactate dehydrogenase.

*Patients who had been followed for more than 6 months and who did not receive any hematopoietic stem cell transplantation were analyzed.

In FL patients, the relapse rate was high in patients with low plasma miR-92a in CR + CRu (5/8 versus 1/11; *P* = .013). The odds ratio for relapse in the low plasma miR-92a group was 16.67 (95% CI: 1.361 to 204.2), but other markers were not statistically significant (**Table 2**). These data suggest the possibility that plasma miR-92a levels could be a biomarker to predict relapse, even in patients with clinically determined CR or CRu in FL. Multivariate analysis demonstrated that a low plasma miR-92a level did not reach statistical significance in predicting relapse owing to the insufficient number of examined patients (data not shown).

**Table 2:** Statistical analysis of relapse in follicular lymphoma patients whose miR-92a levels were determined at complete response and complete response uncertain phase.

			univariate
Factors at diagnosis	No. (n = 19)	Relpased (n = 6)	*P* value	odds ratio	95% CI
Sex					
male	10	3	.876	0.857	0.1235 to 5.947
female	9	3			
Age					
≥60 years	9	4	.252	3.2	0.4192 to 24.43
<60 years	10	2			
LDH					
Elevated	2	0	.31	0.354	0.01463 to 8.559
Normal	17	6			
Hemoglobin					
≤120 g/L	3	2	.154	6	0.4220 to 85.30
≥120 g/L	16	4			
Ann Arbor stage					
III+IV	13	5	.342	3.125	0.2776 to 35.18
I+II	6	1			
No. of nodal area involved					
>4	6	2	.911	1.125	0.1425 to 8.884
≤4	13	4			
Bone marrow invasion					
Yes	8	3	.636	1.6	0.2271 to 11.27
No	11	3			
β2-microglobulin elevation*					
Yes	8	3	.41	2.4	0.2910 to 19.79
No	10	2			
FLIPI					
High	3	1			
Intermediate	8	3	.863		
Low	8	2			
FLIPI-2*					
High	4	3			
Intermediate	11	2	.047		
Low	3	0			
Plasma miR-92a					
Low (<0.2165)	8	5	.013	16.67	1.361 to 204.2
Normal (≥0.2165)	11	1			

Abbreviations: CI, confidence interval; LDH, lactate dehydrogenase; FLIPI, Follicular Lymphoma International Prognostic Index.

*Data from 1 patient were missing.

### MiR-92a expression in lymphoma tissue

**Figure 4:**
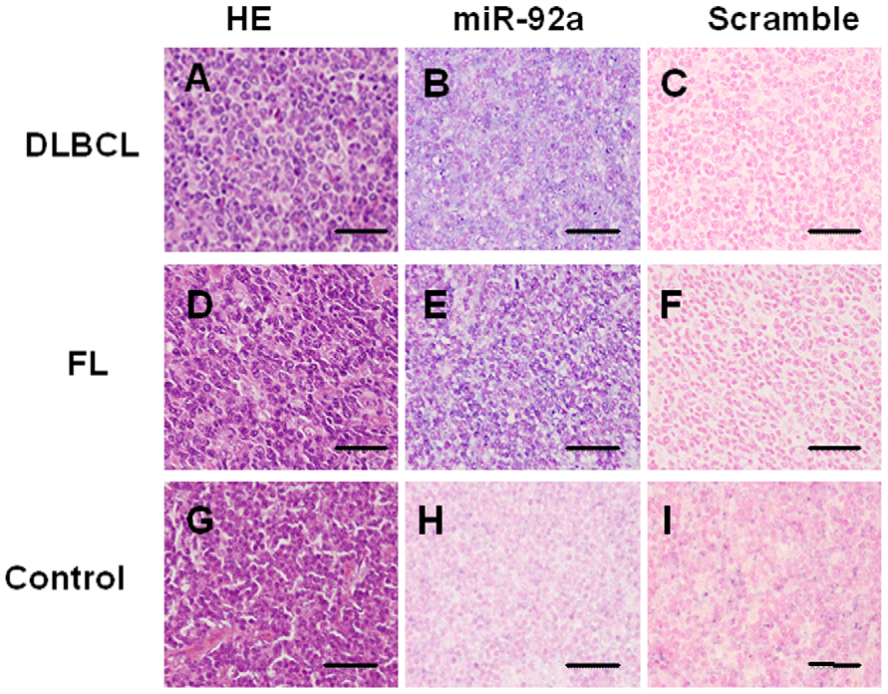
*In situ* MiRNA expression in malignant lymphoma tissues. (A), (B) and (C) were diffuse large B-cell lymphoma. (D), (E) and (F) were follicular lymphoma. (G), (H) and (I) were normal lymph node. (A), (D), (G), were H&E staining. (B), (C), (E), (F), (H) and (I) were *in situ* hybridization LNA probes for miR-92a and in the negative control. Blue signals (BCIP/NBT) represent positive results for miR-92a. Nuclear staining (counter stains) was nuclear fast red. We found positive signals for miR-92a in both nucleus and cytoplasm in (B) and (E). We could not detect positive signals in (G) and scrambled control (C), (F) and (I). Bars indicate 50 µm.

We also examined the expression levels of miR-92a in lymphoma tissues. We performed in situ hybridization using LNA-modified probes labeled with digoxigenin. We examined 2 cases each of DLBCL and FL, and found that miR-92a was strongly expressed in lymphoma cells from both DLBCL and FL. However, miR-92a expression was not detected in lymph nodes obtained from non-lymphoma patients (controls) (**Figure 4**).

## Discussion

Detection of miRNAs in human plasma has recently been demonstrated in healthy subjects, as well as in cancer-bearing patients [21]–[27]. Mitchell et al. clearly demonstrated that miRNAs are present in a stable form in human plasma and serum, and miR-141 originating from human prostate cancer showed the greatest differential expression in prostate cancer serum specimens [21]. Therefore, elevation of certain circulating miRNAs could indicate the presence of certain cancers. Recently, Lawrie et al. also reported that various miRNAs, including miR-155, miR-210, and miR-21, were elevated in the serum of DLBCL patients, and that miR-21 expression was associated with relapse-free survival [25].

Amplification and over-expression of cellular miR-17-92 has been observed in hematologic malignancy, including B-cell lymphoma [28]–[30], T-cell acute lymphoblastic leukemia [31], and purified chronic myeloid leukemia cells [32]. In the current study, however, we found extremely low plasma levels of miR-92a in lymphoma patients compared with healthy individuals. The reduced plasma miR-92a levels partially recovered after CR was obtained but were decreased again in the relapse phase, suggesting that plasma miR-92a levels correlate with disease conditions in lymphoma patients. The changes in plasma miR-92a levels in various clinical states were also inversely correlated with β2-microglobulin or lactate dehydrogenase levels (data not shown). In the present series of lymphoma tissues, however, over-expression of miR-92a was clearly shown using in situ hybridization. Therefore, discrepancy of miR-92a levels between lymphoma tissues and plasma might be of great import. In addition, we found that miR-92a plasma levels in lymphoma patients were less than 5% that of healthy subjects, although the exact mechanism of down-regulation of miR-92a in cancer-bearing patients is still obscure. Shigoka et al also demonstrated the discrepancy of miR-92a levels between tumor tissues and plasma in patients with hepatocellular carcinoma [27].

Recently, it has been demonstrated that tumor-bearing hosts down-regulate miR-17-92 in CD4^+^ T cells, and it has been postulated that a type-2-skewing tumor microenvironment induces the down-regulation of miR-17-92 expression in T cells; CD4+ T cells from patients with glioblastoma multiforme had extremely low levels of 17-92 polycistronic miRNAs [33], suggesting the possibility that the low plasma miR-17-92 level in lymphoma patients may reflect miR-17-92 levels in host T cells. Circulating miRs could be taken up and could function in the recipient cells, including intercellular immune communication [34], [35]. Because some of our NHL patients who obtained CR did not show normalization of plasma miR-92a levels and exhibited a significantly high relapse rate, it is likely that low plasma miR-92a levels may correlate with failure of anti-tumor immune responses. In contrast, over-expression of miR-92a in lymphoma cells may enhance neo-angiogenesis indirectly [16], while angiogenesis progresses under conditions of reduced miR-92a levels in endothelial cells [18]. Therefore, down-regulation of plasma miR-92a levels is also possible and may be due to exosomal transport to vascular endothelial cells. Most recently, Fichtlscherer et al found that circulating levels of vascular (miR-126, miR-17 and miR-92a) and inflammation-associated miRNA (miR-155) were significantly reduced in patients with coronary artery disease [36]. Nevertheless, plasma miR-92a level seems to be unrelated to lymphoma burden, because there was no significant difference among clinical stages and miR-92a values in NHL patients.

Another possibility of down-regulation of plasma miR-17-92 in lymphoma patients might be due to excess degradation of miR-17-92 in plasma or epigenetic silencing [37], [38], however, we have no evidence for these possibilities in cancer-bearing patients.

The extremely low plasma miR-17-92 level might be a representative reaction of lymphoma-bearing patients and is associated with the hematologic condition. The clinically important message is that low levels of plasma miR-92a in CR status are linked to relapse. In the current study, low levels of plasma miR-92a in the CR phase with NHL were related to a significantly high relapse rate, suggesting that plasma miR-92a levels could be used to monitor disease condition. This evidence is particularly clear in FL and high-grade DLBCL patients. Although the number of patients in this series is still small, the results may be useful in determining a strategy for second-line therapy for NHL patients. Therefore, a combination of classic anatomical assessment and miR-92a levels could be a powerful tool for monitoring disease status. Additional information regarding plasma miR-17-92 expression may emerge from studies of the pathophysiology of the immune response of cancer-bearing patients and may also provide a promising approach for immunotherapy or anti-neo-angiogenesis therapy for cancer patients.

## Materials and Methods

### Patients and samples

We evaluated peripheral blood obtained from 126 patients (144 specimens) with non-Hodgkin's lymphoma (NHL) after obtaining their written informed consent: 76 patients (87 specimens) had diffuse large B-cell lymphoma (DLBCL), 32 patients (36 specimens) had follicular lymphoma (FL), and 18 patients (21 specimens) had T-cell NHL. Of the 87 samples from patients with DLBCL, 32 samples were studied at the time of diagnosis, 9 at relapse, and 46 at the complete remission (CR) phase. Of the 46 samples, 11 patients were analyzed at both diagnosis and the CR phase. The 36 specimens from patients with FL consisted of 12 samples at diagnosis, 11 at CR, 8 at CR unconfirmed (CRu), and 5 at relapse; 4 patients were studied at both diagnosis and CR/CRu. Of the 21 samples from patients with T-cell NHL, 16 samples were studied at the time of diagnosis, 2 at relapse, and 3 at the CR/CRu phase; 3 patients were analyzed at both diagnosis and the CR phase (**Table S1**). Patients were treated with standard chemotherapy, for example, rituximab plus CHOP or CHOP-like regimens for CD20-positive NHL and CHOP-based therapy for T-cell NHL. Complete remission (CR) or CR unconfirmed (CRu) status was evaluated by standard criteria [39], [40]. For relapsed statistical analysis, we utilized plasma from selected patients (53 out of 65 patients with CR + CRu) who had been followed-up for more than 6 months after chemotherapy without any hematopoietic stem cell transplantations. This study was approved by the institutional review board of Tokyo Medical University (no. 930; approved on June 24, 2008). Written informed consent was obtained from all patients according to the Declaration of Helsinki prior to collection of the specimens.

Total RNA in the plasma was isolated by using Isogen-LS (Nippon Gene, Tokyo, Japan) according to the manufacturer's instructions. For RNA isolation from plasma, 250 µl of plasma was homogenized in 750 µl of Isogen-LS, then 200 µl of chloroform was added to the sample, and the mixed solution was centrifuged. After additional chloroform extraction and precipitation with isopropanol, the RNA sample was suspended in 20 µl of nuclease-free water [26]. Usually, we obtained about 300 ng of RNA from 250 µl of plasma.

### MiRNA microarray analysis

To monitor the changes in miRNA levels associated with NHL, we labeled and hybridized 100 ng of total RNA using the Human microRNA Microarray Kit (Agilent Technologies, Wilmington, DE, USA) according to the manufacturer's protocol (Protocol for Use with Agilent MicroRNA Microarrays Version 1.5). Hybridization signals were detected with a G2505C microarray scanner (Agilent Technologies), and the scanned images were analyzed with Agilent feature extraction software 10.7.0. according to the default protocol [26] All data is MIAME compliant, and the data discussed in this manuscript have been deposited in NCBI's Gene Expression Omnibus and are accessible through GEO Series accession number GSE22604: http://www.ncbi.nlm.nih.gov/geo/query/acc.cgi?token=jxkzbkecqkqscdy&acc=GSE22604.

### Quantitative real-time PCR of mature miRNAs

We quantified miRNAs using TaqMan® MicroRNA Assays (Applied Biosystems, Foster City, CA, USA) with modifications [26] and miRNA-specific stem-loop primers (has-miR-92a, 000431; has-miR-638, 001582; Applied Biosystems). Subsequently, quantitative real-time polymerase chain reaction (PCR) was performed with an ABI Prism 7000 sequence detection system (Applied Biosystems). The reaction was initiated by incubation at 95°C for 2 min, followed by 50 cycles of 95°C for 15 sec and then 60°C for 1 min. All reactions were run in duplicate. Mean cycle threshold (C_t_) values for all miRNAs were quantified with sequence detection system software (SDS Version 1.02; Applied Biosystems). The miR-92a expression was normalized to miR-638 expression, yielding a –ΔC_t_ value. The –ΔΔC_t_ value was then calculated by subtracting the –ΔC_t_ value of a normal sample from the respective –ΔC_t_ values of the patient samples. Expression of miR-92a was normalized by using the 2^-ΔΔCt^ method [26], [27].

We also analyzed plasma miR-17-92a expression using TaqMan® MicroRNA Assays (Applied Biosystems) and miRNA-specific stem-loop primers (has-miR-17, 002308; has-miR-18a, 002422; has-miR-19a, 000395; has-miR-19b, 000396; and has-miR-20a, 000580 [Applied Biosystems]).

### MiR-92a expression in the lymphoma cells

Locked nucleic acid (LNA)-modified probes for miR-92a and negative control (miRCURY-LNA detection probe, Exiqon, Vedbaek, Denmark) were used to detect miR-92a expression in biopsied lymph nodes. The probe sequences were as follows: *miR-92a*, *5′-ACAGGCCGGGACAAGTGCAATA-3′*, scrambled oligonucleotides used for the negative control, *5′-GTGTAACACGTCTATACGCCCA-3′*. In situ hybridization was performed with the RiboMap in situ hybridization kit (Ventana Medical Systems, Tucson, AZ, USA) on a Ventana Discovery automated in situ hybridization instrument (Ventana Medical Systems) [26], [27].

### Statistical analysis

We used GraphPad Prism 5.0 software (GraphPad Software Inc., San Diego, CA, USA) for statistical analysis. The Mann-Whitney and chi-square tests were used to determine statistical significances between the control and test groups. Multivariate analysis was performed with Excel Multivariate Analysis Version 5.0 (Esumi KK, Tokyo, Japan). *P* values less than.05 were considered to indicate statistically significant differences.

## Supporting Information

Table S1 **Plasma miR-92a/miR-638 expression levels in non-Hodgkin's lymphoma.**
(DOC)
