# Supplementary material for: Clinical Impact of Down-Regulated Plasma miR-92a Levels in Non-Hodgkin's Lymphoma
Source: PLoS One. 2011 Feb 24;6(2):e16408. doi: 10.1371/journal.pone.0016408 (PMC3044711; doi:10.1371/journal.pone.0016408)
Supplement: Supplementary file 1 [file pone.0016408.s001.doc]

Table S1: Plasma miR-92a/miR-638 expression levels in non-Hodgkin's lymphoma

	No. of patients	Median	Standard error	Minimum	Maximun	95% confidence interval
Healthy volunteers	37	1.000	0.1508	0.2154	3.580	0.8939 to 1.506
Diffuse large B-cell lymphoma						
at diagnosis	32	0.03013	0.01570	0.0001058	0.3789	0.03689 to 0.1009
Stage 1	5	0.07056	0.03800	0.005048	0.1928	-0.02023 to 0.1908
Stage 2	5	0.04688	0.06924	0.0001058	0.3789	-0.08189 to 0.3026
Stage 3	7	0.04704	0.02624	0.008004	0.2176	0.007729 to 0.08841
Stage 4	15	0.01346	0.01881	0.003002	0.2717	0.007729 to 0.08841
Relapse	9	0.03678	0.02260	0.003129	0.2207	0.007475 to 0.1117
Complete response	46	0.3424	0.08256	0.03202	1.840	0.3799 to 0.7124
Follicular lymphoma						
at diagnosis	12	0.02547	0.01814	0.006108	0.2373	0.006179 to 0.08605
Relapse	5	0.05292	0.01235	0.03315	0.1026	0.02271 to 0.09130
Complete response	11	0.3610	0.1881	0.1055	2.211	0.1725 to 1.011
Complete response uncertain	8	0.09542	0.08919	0.02164	0.7792	0.02271 to 0.09130
T-cell lymphoma						
at Diagnosis	16	0.07647	0.01704	0.002810	0.2293	0.05921 to 0.1318
Complete response (CR+CRu)	3	2.761	0.8305	1.046	3.903	-1.003 to 6.144
Relapse	2	0.3103	0.2741	0.03615	0.5844	-3.173 to 3.793

Abbreviations: CR, complete response; CRu, complete response uncertain.
